# Self‐reported stress, coping ability, mental status, and periodontal diseases among police recruits

**DOI:** 10.1002/cre2.258

**Published:** 2019-11-24

**Authors:** Shaun Ramlogan, Vidya Raman, Kimberly Abraham, Kereesa Pierre

**Affiliations:** ^1^ Periodontology, Restorative Unit, School of Dentistry University of the West Indies Trinidad and Tobago

**Keywords:** mental status, periodontitis, plaque score, stress, stress coping

## Abstract

**Objective:**

This cross‐sectional study aimed to investigate self‐reported stress level and coping ability as well as mental status (anxiety and depression) via the 12‐item General Health Questionnaire (GHQ‐12) questionnaire and periodontal status among police academy recruits during their 8 months of training.

**Methods:**

Eighty‐five consenting police recruits were examined at baseline during the first month of training and again during the last month of training. Full mouth plaque score (FMPS), full mouth bleeding score, basic periodontal examination, self‐reported stress level (scale of 1–10) and GHQ‐12 questionnaire (mental status) were recorded at both visits. Ability to cope (yes/no) with stress was recorded at the final visit. Periodontal diagnosis was derived based on clinical examination. *t* test and regression analyses (*p* < .05) were performed.

**Results:**

High stress (odds ratio: 1.25) and inability to cope with stress (odds ratio: 1.31) were statistically significant (*p* < .05) predictors of high FMPS. Inability to cope with stress (odds ratio: 1.45) was also a statistically significant (*p* < .05) predictor for periodontitis compared to gingivitis. Mental status (anxiety and depression) may play a greater role in gingivitis (mean 1.75) as opposed to periodontitis (mean 1.00) as reflected by the higher mean GHQ‐12 (*t* test, *p* = .04).

**Conclusions:**

In this study, both self‐reporting of stress level and ability to cope with stress were statistically significant predictors of higher plaque score (FMPS). Ability to cope with stress was also a statistically significant predictor of periodontitis compared to gingivitis. Recording of both self‐reported stress level and ability to cope may be valuable variables to note in the management of plaque and periodontal diseases.

## 1 INTRODUCTION

Periodontal diseases of gingivitis and periodontitis are inflammatory diseases involving the periodontium with an aetiology of plaque but with many multifactorial modulating factors. One of these modulating factors, psychosocial stress, is the effect of both psychological (behaviour of the mind) and social environmental factors that result in strain and distress on the physical and mental well‐being of an individual. Psychosocial stress has been shown to have both psychological behavioural changes, such as smoking and poor oral hygiene habits, and physiological effects, such as changes in immune response and wound healing (Deinzer, Hilpert, Bach, Schawacht, & Herforth, 2001; Genco et al., 1998; Rozlog, Kiecolt‐Glaser, Marucha, Sheridan, & Glaser, 1999). One study reported an increase in proinflammatory mediators at chronic inflammation sites of the gingiva in response to an episode of acute stress (Weik, Herforth, Kolb‐Bachofen, & Deinzer, 2008).

The effect of exam stress on medical students was related to higher levels of plaque among those with exams as opposed to matched controls without exams (Deinzer et al., 2001). There was an increase in gingival inflammation as measured by bleeding on probing in students stressed by exams in comparison to matched controls without exams over a 4‐week period (Deinzer, Rüttermann, Möbes, & Herforth, 1998). The psychological and immunological effects on stressed individuals may also have a time dissociation with the latter demonstrating a prolonged effect after the episode of stress (Deinzer, Kleineidam, Stiller‐Winkler, Idel, & Bachg, 2000). Financial stress and also poor coping skills have been associated with an increased risk for periodontal diseases (Genco, Ho, Grossi, Dunford, & Tedesco, 1999).

Stress may produce changes in mental and psychological well‐being to include signs of depression, anxiety, and altered self‐esteem. In an animal model, depression accelerated periodontal tissue breakdown and was reversed by antidepressive drug therapy (Breivik et al., 2006). However, whereas some human studies link anxiety and depression with periodontal diseases, others fail to show a relationship. A systematic review of stress and psychological factors as a risk factors for periodontal diseases revealed a positive outcome in 57%, equivocal results for 28%, and negative outcome for 14% involving 14 included studies (Peruzzo et al., 2007).

The General Health Questionnaire (GHQ) is a quantitative self‐reported questionnaire method developed to determine risk for psychiatric disorders (Goldberg & Blackwell, 1970). The GHQ was reported as a commonly validated self‐reported method in systematic reviews of anxiety or depression measures (Hewitt, Perry, Adams, & Gilbody, 2011; Meades & Ayers, 2011). The original version of 60 questions (GHQ‐60) has been proposed in a shortened form for ease of applicability to 12 questions (GHQ‐12) by Golderberg and Williams (1988) The GHQ‐12 questionnaire excluded questions that were endorsed by the physically ill from the original GHQ‐60 version. The GHQ‐12 has been shown to have good reliability in the general population (Petkovska, Bojadziev, & Stefanovska, 2015) and further validity for measures of anxiety and depression (Baksheev, Robinson, Cosgrave, Baker, & Yung, 2011; Lundin, Hallgren, Theobald, Hellgren, & Torgén, 2016).

Many studies have employed structured questionnaires to measure stress and psychological status (Peruzzo et al., 2007). Additionally, comparisons are made with groups exposed to stress and those not exposed to stress rather than looking at the experience of all individuals within a group with similar environmental exposure (Deinzer et al., 1998; Deinzer et al., 2001). A simpler form of stress quantification such as the self‐reported stress levels on a scale of 1–10 has ease of application to the clinical setting and has had little reporting in the literature. The relationship between stress and periodontal diseases continues to be an underscored area of study that requires further investigation.

Thus, the aim of this study was to investigate self‐reported stress levels and coping ability as well as associated mental status (anxiety and depression) via the GHQ‐12 questionnaire and periodontal status in a group of police academy recruits during their training programme.

### 1.1 Study population and methodology

Ninety‐six police recruits from the class of 2018, Police Academy, Trinidad and Tobago Police Service, were invited to participate in this cross‐sectional study. All academy recruits were included without exclusion criteria once consent to participate was obtained to eliminate selection bias for stress, mental status, and periodontal disease. Selection of police recruits also reduced confounding variables of systemic disease as these recruits were expected to be reasonably fit and healthy. The duration of the training course was a period of 8 months. Ethical approval was obtained from the University of the West Indies Ethics Committee (Ref: CEC400/11/17).

**Figure 1:**
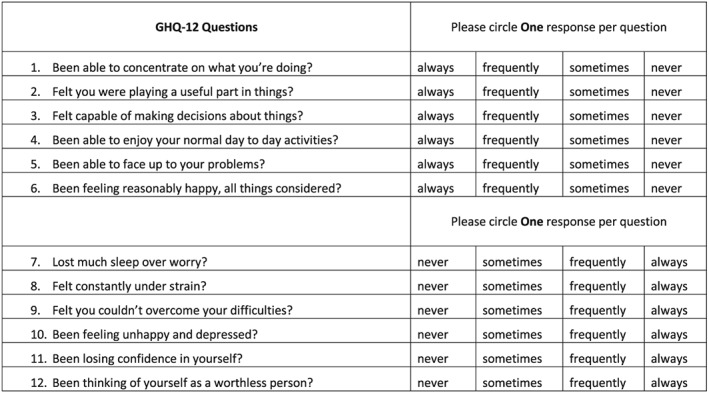
Twelve‐item General Health Questionnaire (GHQ‐12)

This study composed of a demographic and health questionnaire that was completed by an interviewer and covered medical and dental histories to include self‐reported anxiety (yes/no) and depression (yes/no) and temporomandibular complex problems (yes/no). Self‐reported stress levels on a scale of 1–10 with 10 being the highest stress level were recorded. Psychological well‐being or mental status was determined by the GHQ‐12 questionnaire that was completed unaided by participants. The GHQ‐12 consisted of six positive questions and six negative questions with a 4‐point Likert scale response from always to never (see Figure 1). However, a binominal 0,0,1,1 scoring was applied from always to never for positive questions and never to always for negative questions. This was recommended to reduce the bias in response (Golderberg & Williams, 1988). Thus, there was a total maximum score of 12 for all questions indicating the worse mental well‐being status.

The questionnaire was followed by a clinical dental examination that composed of a periodontal assessment via basic periodontal examination (BPE) scoring, full mouth plaque score (FMPS), and full mouth bleeding score (FMBS (Ainamo & Bay, 1975; The British Society of Periodontology (BSP), Council of the BSP, n.d.);). All patients were examined by one examiner who was a periodontologist. In the absence of dental radiological services, a tentative periodontal diagnosis in general terms of either healthy, gingivitis, or periodontitis was assigned based on clinical gingival presentation, FMPS, FMBS, BPE codes, interproximal recession, and attachment loss. In determining a diagnosis, care was taken to account for any false pockets (i.e., free gingival margin above the cementoenamel junction), and thus, patients with a true BPE of 4 were ascribed a periodontitis diagnosis. Additionally, patients with a BPE of 3 and interproximal recession were also assigned a diagnosis of periodontitis. In the periodontal diagnosis, further description of distribution or severity of the periodontal diseases was not attempted.

This first visit occurred within the first month of the training programme to determine the baseline scores of all recruits. All cases were coded via identification numbers to maintain confidentiality. The recruits were revisited within the last month of the training programme approximately 6 months later. At this final visit, the examiner was blind to the previously collected data. Self‐reported stress levels, ability to cope with stress, GHQ‐12 questionnaire, and repeat periodontal indices of FMPS, FMBS, and BPE were obtained at this final visit. Recruits were afforded individual feedback and group dental education only at this visit so as not to influence the outcome.

Self‐reported stress levels were categorized as low for 1 to 5 and high for 6 to 10. Specific cohort‐based GHQ‐12 low and high categories were also derived based on a cut‐off value established by the mean for the whole group (Goldberg, Oldehinkel, & Ormel, 1998). IBM SPSS Statistics 24 statistical software (IBM Corporation, Armonk, NY, USA) was used in the analyses. *t* test (*p* < .05) comparisons of the mean periodontal indices of FMPS, FMBS, and highest BPE code in the (a) low stress and high stress level group, (b) inability to cope and ability to cope with stress groups, and (c) low and high GHQ‐12 were derived. Regression analyses were conducted for the indices, which showed a statistical difference to account for confounding variables. *t* test (*p* < .05) comparisons of the mean stress level and mean GHQ‐12 values as grouped by diagnoses were also derived.

## 2 RESULTS

There were 85 recruits (88.5%) who consented to participate in this study out of a total class of 96 individuals. Seventy‐four percent (*n* = 63) were male, and 26% (*n* = 22) were female. The mean age was 25.1 years (standard deviation: 4.4, range: 19–35 years). With regard to marital status, the majority of recruits were single at 69.4% (*n* = 59), and the remainder of 30.6% (*n* = 26) was in some form of relationship (married or partner). Only 1 (1.2%) of the 85 recruits was a current smoker. Nine (10.6%) recruits had some significant medical history (asthma, *n* = 6; high blood pressure, *n* = 2; minor heart condition, *n* = 1). There was an attrition rate of approximately 10% as eight recruits failed to return for the second visit due to study and training commitments.

All mean periodontal indices (FMPS, FMBS, and BPE) except the final mean BPE were higher for the high stress level group (stress: 6–10) compared with the low stress level group (stress: 1–4) for both the initial and final visits. There was no statistically significant difference between the periodontal indices for the stress level groups at the initial visit. The values are shown in Table 1 for the final visit with only the final mean FMPS being statistically significantly higher (*t* test, *p* = .004) for the high (mean: 16.00, standard deviation: 9.95) compared with the low (mean: 10.44, standard deviation: 5.70) stress level group. Further, when retaining these stress level category groups from the final visit, there was a lack of statistical difference (*t* test, *p* = .68) between the initial mean FMPS for high stress level (mean: 14.56, standard deviation: 12.94) and the low stress level (mean: 13.47, standard deviation: 8.68) groups at the first visit.

**Table 1:** Comparison of mean periodontal indices versus stress level category at final visit

Periodontal index	Stress level category	*N*	Mean	Standard deviation	*t* test sig.
FMPS	Low	59	10.44	5.70	<.01
	High	18	16.00	9.95	
FMBS	Low	59	11.49	5.98	.94
	High	18	11.61	4.64	
BPE	Low	59	2.08	0.82	.55
	High	18	1.94	1.00	

Abbreviations: BPE, basic periodontal examination; FMBS, full mouth bleeding score; FMPS, Full mouth plaque score.

The mean stress level for those recruits diagnosed with gingivitis (*n*: 65, mean: 5.15, standard deviation: 2.27) was higher than those diagnosed with periodontitis (*n*: 17, mean: 4.82, standard deviation: 2.13) at the initial visit. This difference was not statistically significantly different (*p* = .58). Recruits reporting signs of temporomandibular joint complex problems had a statistically significantly (*t* test; *p* = .046) higher mean stress levels (*n*, mean, standard deviation; 15, 6.13, 2.36) compared with the mean stress levels (*n*: 67, mean: 4.87, standard deviation: 2.15) of those without problems.

Mean stress levels for all recruits were not statistically different (paired *t* test, *p* = .06) between initial (mean: 5.06, standard deviation: 2.20) and final (mean: 4.58, standard deviation: 2.11) visits despite the reportedly lower stress levels at the final visit. There was a lack of statistically significant difference (paired *t* test, all *p* > .05) between the initial and final periodontal indices despite a trend for lower FMPS and FMBS and higher BPE at the final visit compared with initial visit (Table 2) for all recruits.

**Table 2:** Comparison of initial and final mean periodontal indices

Periodontal index	Initial mean values (standard deviation) *n* = 85	Final mean values (standard deviation) *n* = 77	Paired sample *t* test sig.
FMPS	13.7 (9.7)	11.6 (7.3)	.07
FMBS	12.1 (6.0)	11.5 (5.7)	.40
BPE	1.8 (0.8)	2.0 (0.9)	.10

Abbreviations: BPE, basic periodontal examination; FMBS, full mouth bleeding score; FMPS, full mouth plaque score.

In comparison of mean periodontal indices with ability to cope with stress, all mean periodontal indices were higher for those who could not cope compared with those who could cope. The mean values were statistically significantly different for both FMPS and BPE (*p* = .03) but not for FMBS (Table 3).

**Table 3:** Comparison of mean periodontal indices with ability to cope with stress at final visit

Periodontal index	Coping with Stress	*N*	Mean	Standard deviation	*t* test sig.
FMPS	Yes	40	10.03	5.39	.03
	No	37	13.57	8.54	
FMBS	Yes	40	10.40	4.67	.08
	No	37	12.73	6.43	
BPE	Yes	40	1.85	0.66	.03
	No	37	2.27	0.99	

Abbreviations: BPE, basic periodontal examination; FMBS, full mouth bleeding score; FMPS, full mouth plaque score.

Table 4 shows the comparison of mean GHQ‐12 values for recruits with gingivitis and periodontitis at the initial and final examinations with exclusion of the three healthy patients for analysis due to the small number. There was a statistically significantly (*t* test, *p* = .04) larger mean GHQ‐12 value for the gingivitis group (1.75) compared with the periodontitis group (1.00) at the initial visit. However, the final visit failed to show this trend or attain statistical significance.

**Table 4:** Comparison of mean GHQ‐12 for gingivitis and periodontitis recruits

Session	Diagnosis	N	Mean	Std. Deviation	*t* test sig.
Initial GHQ‐12	Gingivitis	65	1.75	1.86	.04
	Periodontitis	17	1.00	1.06	
Final GHQ‐12	Gingivitis	59	1.07	1.47	.88
	Periodontitis	15	1.13	1.77	

Abbreviation: GHQ‐12, 12‐item General Health Questionnaire.

Study specific categories of low (<2) and high (≥2) GHQ‐12 were derived based on the mean GHQ‐12 value for all recruits. Comparison of mean periodontal indices of FMPS, FMBS, and BPE revealed higher values for all indices for the high GHQ‐12 versus the low GHQ‐12 categories at both initial and final visit. However, none of these differences for the mean periodontal indices were statistically significant (*t* test, all *p* > .05).

Table 5 shows the comparison of the mean GHQ‐12 values for recruits with self‐reported anxiety and depression for the initial visit. The group of persons reporting the presence of anxiety or depression had statistically significantly (*t* test, *p* < .01) higher mean GHQ‐12 values compared with the group without anxiety or depression.

**Table 5:** Comparison of mean GHQ‐12 for recruits with anxiety or depression at initial visit

Session	Mental state		*N*	Mean	Standard deviation	*t* test sig.
Initial GHQ‐12	Anxiety	Yes	9	3.67	2.35	<.01
		No	76	1.30	1.48	
	Depression	Yes	8	3.25	2.91	<.01
		No	77	1.38	1.48	

Abbreviation: GHQ‐12, 12‐item General Health Questionnaire.

The mean GHQ‐12 value was statistically significantly higher (*t* test, *p* = .01) at 2.11 (standard deviation: 2.07) for the high stress level group compared with the mean of 1.14 (standard deviation: 1.32) for the low stress level group at the initial visit. There was also statistically significant correlation (Pearson's correlation coefficient: .41; *p* < .01) between the initial GHQ‐12 value and the initial stress level. However, comparisons at the final visit between the GHQ‐12 for the high and low stress level category were not statistically significantly different.

Overall the recruits showed a decrease from the initial mean GHQ‐12 value of 1.47 (standard deviation: 1.55) to the final mean GHQ‐12 value of 1.06 (standard deviation: 1.51). The difference in the means was statistically significant using paired *t* test (*p* = .03).

Regression model statistical analyses with dependent variable of FMPS and predictor variables of gender, age, relationship status, smoker, significant medical history, floss use, visit to the dentist, stress level, stress coping, and GHQ‐12 score are shown in Table 6. Relationship status of married or partnered (odds ratio: 0.77), lack of use of floss (odds ratio: 1.42), high stress levels (odds ratio: 1.25), and inability to cope with stress (odds ratio: 1.31) were statistically significant (*p* < .05) predictors of higher FMPS.

**Table 6:** Regression model with dependent variable of FMPS

Predictor variables	Odd ratio exp (B)	95% confidence interval of odds ratio (lower and upper value)	Sig.
Gender	0.94	[0.05, 1.19]	.60
Age	1.19	[0.94, 1.50]	.16
Relationship status	0.77	[0.61, 0.97]	.03
Smoker	0.95	[0.75, 1.19]	.64
Medical history	0.96	[0.78, 1.20]	.74
Floss	1.42	[1.11, 1.81]	<.01
Visit dentist	0.86	[0.68, 1.09]	.21
Stress level	1.25	[1.01, 1.56]	<.05
Stress coping	1.31	[1.04, 1.66]	.02
GHQ‐12	1.15	[0.68, 1.93]	.61

Abbreviation: FMPS, full mouth plaque score; GHQ‐12, 12‐item General Health Questionnaire.

Regression model statistical analyses were also obtained with dependent variable of periodontal diagnosis and predictor variables of gender, age, relationship status, smoker, significant medical history, FMPS, visit to the dentist, stress level, stress coping, and GHQ‐12 score as shown in Table 7. Female gender (odds ratio: 1.32), married/partnered relationship status (odds ratio: 0.77), high FMPS (odds ratio: 1.45), and inability to cope with stress (odds ratio: 1.45) were statistically significant (*p* < .05) predictors of a periodontitis compared with gingivitis diagnosis.

**Table 7:** Regression model with dependent variable of periodontal diagnosis

Predictor variables	Odd ratio exp (B)	95% confidence interval of odds ratio (lower and upper value)	Sig.
Gender	1.32	[1.05, 1.67]	.02
Age	1.06	[0.84, 1.33]	.60
Relationship status	0.77	[0.61, 0.97]	.03
Smoker	0.93	[0.74, 1.17]	.54
Medical history	0.88	[0.71, 0.91]	.25
FMPS	1.45	[1.11, 1.70]	<.01
Visit dentist	1.12	[0.92, 1.36]	.27
Stress level	0.98	[0.78, 1.24]	.89
Stress coping	1.45	[1.15, 1.82]	<.01
GHQ‐12	0.91	[0.72, 1.36]	.42

Abbreviation: FMPS, full mouth plaque score; GHQ‐12, 12‐item General Health Questionnaire.

## 3 DISCUSSION

The mean FMPS was larger with statistical significance (*t* test, *p* = .004) for the high stress level group compared with the low stress level group at the final visit. By comparison, the baseline mean FMPS was not statistically significantly different for the same individuals in the high stress level compared with the low stress level group categories at the initial visit. Additionally, the entire group of recruits did not demonstrate a change with statistical significance in mean FMPS or stress level between the initial visit and final visit.

Thus, the high stress level recruits had significantly higher accumulations of plaque as measured by the FMPS at the end compared with the beginning of the training programme. This finding agrees with another study on stress and FMPS (Deinzer et al., 2001). The higher FMPS may be postulated for by changes in oral hygiene habits and oral physiology (e.g,. saliva flow and immunology) among the high stress group. The lack of statistical difference in FMPS between the two stress level groups initially may reflect the more dynamic nature of stress and the temporal relationship (Deinzer et al., 2000) to the outcome. Despite recruits reporting high stress levels at baseline, there was no difference in FMPS compared with the low stress level counterparts. However, after experiencing a stress challenge, the high stress level group demonstrated a statistically significant change in their FMPS.

Coping with stress was only indicated at the final visit as a measure of the ability to manage the stress due to the training programme. Recruits who were not able to cope with stress had higher FMPS and BPE with statistical significance (*p* = .03 for both) compared with those who could cope. The authors recognize that the BPE scoring, a continuous limited number range index, does not measure similar equivalent increments as the FMPS and FMBS. However, it was included to reflect a gradation or measurement of increasing periodontal involvement. The lack of statistically significant difference for the FMBS between groups for coping with stress may be a reflection of the temporal limitations. Changes in bleeding may lag behind plaque accumulation. Another reason for the lack of difference with FMBS may be localization of changes and varying susceptibility of each recruit to periodontal disease. Additionally, looking at the low value of *p* = .08 for FMPS, it may also be postulated that the lack of significance may also be due to the low number of participants in this study with a larger population potentially revealing a significant result.

Initial GHQ‐12 scores showed high correlation (Pearson's coefficient: .41; *p* < .01) with statistical significance with the initial stress level scores. Likewise, the high stress group had higher mean GHQ‐12 scores compared with the lower stress group with statistical significance (*p* = .01) at the initial visit. These two findings were not demonstrated in the final visit. Further recruits reporting anxiety or depression had higher mean GHQ‐12 scores with statistical significance (*t* test, *p* < .01) at the initial visit. Thus, although GHQ‐12 may be a good measure of anxiety and depression, its relationship with stress may be more complex. Although there was good relationship at baseline between GHQ‐12 and stress, this failed to remain true with the changes at the end of the training programme. It may be assumed that GHQ‐12 may not be sensitive enough to reflect immediate or recent changes in stress.

GHQ‐12 scores were higher with statistical significance *p* = .04 for the gingivitis group compared with the periodontitis group at the initial visit. This may reflect a different nature or role of mental status in recruits with gingivitis compared with periodontitis. This study did not incorporate the new guidelines for defining health and gingivitis from the 2017(Dietrich et al., 2019) classification of periodontal disease because the study protocol was devised prior to its release. The authors also felt the new classification underscored the representation of gingivitis because persons with 32 teeth and 16 sites of bleeding would essentially be healthy. Gingivitis classification in this study included clinical appearance such as erythema, oedema, change in contour, and loss of stippling in addition to bleeding on probing. Additionally, recommendations for periodontitis diagnosis from the 2017 classification support the diagnosis protocol of this paper in that participants with BPE of 3 and interproximal recession represented periodontitis.(The British Society of Periodontology (BSP), n.d.)

All periodontal indices were higher for high versus low GHQ‐12 categories for both initial and final visits. However, these difference did not achieve statistical significance possibly due to drawbacks with scoring, selection of an accurate, or population specific cut‐off point for high and low GHQ‐12 categories and small study sample size. Overall recruits showed a decrease in GHQ‐12 scores from initial to final visit, which achieved statistical significance (paired *t* test, *p* = .03). Likewise, there were decrease in stress levels and periodontal indices of FMPS and FMBS from initial visit to final, but these changes did not achieve statistical significance. This study failed to show a relationship of GHQ‐12 and periodontal indices or diagnosis by regression analyses. The reduction in GHQ‐12 and stress levels at the end of the study was unexpected and may have been due to recruits having less burden of training and exams as the programme was coming to a close.

The impact on periodontal status and stress of the variables of smoking and even significant medical histories was limited due to the small numbers in the latter two groups. This was predictably expected due to the healthy status and requirements for entry into the police academy training programme. Those recruits who self‐reported anxiety and depression were not clinically diagnosed with these conditions and thus were not on any medication to have had an impact on their oral status, stress level, or coping ability.

The variables of married or partnered relationship status (odds ratio: 0.77) or conversely single status (odds ratio: 1.30), lack of use of floss (odds ratio: 1.42), high stress level (odds ratio: 1.25), and inability to cope with stress (odds ratio: 1.31) were statistically significant (*p* < .05) predictors for high FMPS based on regression analyses at the final visit. The variables of female gender (odds ratio: 1.32), relationship (married or partnered; odds ratio: 0.77) or conversely single status (odds ratio: 1.30), and inability to cope with stress (odds ratio: 1.45) were statistically significant (*p* < .05) predictors of worse periodontal diagnosis based on regression analyses. Thus, both simple self‐reporting of stress level (scale of 1–10) and self‐reporting of ability to cope with stress (yes/no) may have a value in predicting plaque scores (FMPS) and may play an important role in management of persons with periodontal diseases.

## 4 CONCLUSION

Self‐reporting stress and ability to cope with stress are valuable factors to note in the prediction of plaque scoring via FMPS and by extension the management of patients with periodontal diseases. This simplified self‐reporting approach has the advantage of being quickly applied and relevant to the current situation of the patient without the need for the detailed and time‐consuming questionnaire methodology. Mental status (anxiety and depression) as measured via the GHQ‐12 questionnaire may play a different role in gingivitis as opposed to periodontitis individuals with a greater effect on the gingivitis patients. However, in this study, mental status was not a significant predictor of periodontal indices or diseases. Further work may be required to compare the mental status in healthy individuals versus individuals with periodontal diseases by including larger study populations and comparisons with other mental health assessment methods.

### 4.1 Clinical relevance

#### 4.1.1 Scientific rational for the study

Stress, coping with stress, and mental status determined via questionnaire has been invariably linked to periodontal disease. Research in this area is historical and limited. Hence, an investigation of a simple self‐reported stress level (Breivik et al., 2006; Deinzer et al., 1998; Deinzer et al., 2000; Deinzer et al., 2001; Genco et al., 1998; Genco et al., 1999; Goldberg & Blackwell, 1970; Peruzzo et al., 2007; Rozlog et al., 1999; Weik et al., 2008) and coping ability (yes/no) as well as associated mental status (anxiety and depression) via the GHQ‐12 questionnaire and periodontal status in a group of stressed police academy recruits was undertaken.

#### 4.1.2 Principal findings

High stress (odds ratio: 1.25) and inability to cope with stress (odds ratio: 1.31) were statistically significant (*p* < .05) predictors of high plaque scores (FMPS). Inability to cope with stress (odds ratio: 1.45) was also a statistically significant (*p* < .05) predictor for periodontitis compared with gingivitis. Mental status (anxiety and depression) may play a greater role in gingivitis compared to periodontitis.

#### 4.1.3 Practical implications

Simple self‐reporting of stress and ability to cope with stress in the clinical setting may be an efficient and quick method of recording factors that significantly impact on plaque scoring and by extension periodontal diseases. Unexpected changes in FMPS and periodontal diseases may be better managed by recognizing the role of stress in disease pathogenesis. Awareness of the level of these two factors (stress level and ability to cope) by both the clinician and the patient may be helpful in the successful management of periodontal diseases.

## CONFLICT OF INTEREST

All authors had no conflict of interest.

## FUNDING INFORMATION

There was no external funding for this project.
